# Falling on deaf neurons

**DOI:** 10.7554/eLife.02289

**Published:** 2014-02-18

**Authors:** Samuel J Sober, Ronald L Calabrese

**Affiliations:** 1 **Samuel J Sober** is in the Department of Biology, Emory University, Atlanta, United Statessamuel.j.sober@emory.edu; 2 **Ronald L Calabrese** is an *eLife* reviewing editor, and is in the Department of Biology, Emory University, Atlanta, United Statesronald.calabrese@emory.edu

**Keywords:** zebra finch, auditory feedback, sensorimotor, birdsong, other

## Abstract

The brain of a bird that is singing appears to be able to block out certain signals and prevent them from influencing brain activity.

**Related research article** Hamaguchi K, Tschida KA, Yoon I, Donald BR, Mooney R. 2014. Auditory synapses to song premotor neurons are gated off during vocalization in zebra finches. *eLife* **3**:e01833. doi: 10.7554/eLife.01833

**Image** False-colour image showing changes in membrane potential as a zebra finch sings

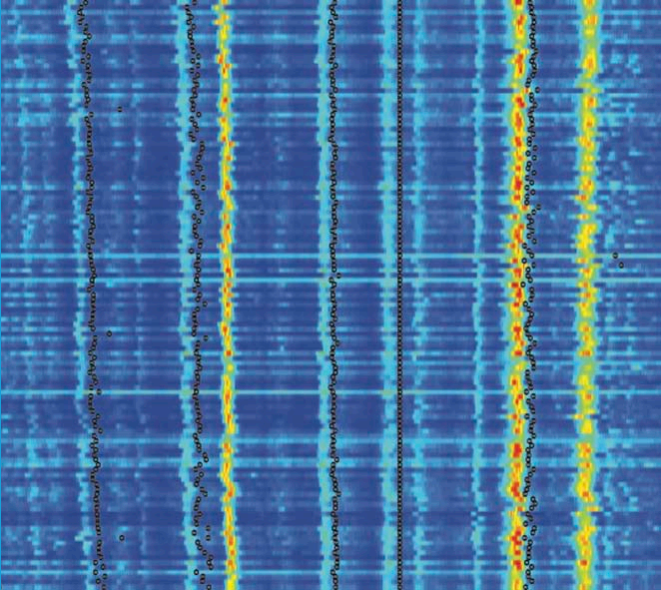

To those of us who live in temperate climes, every spring brings a cacophony of singing birds. To us it is pleasing to the soul; to the birds it is crucial for successful mating. Young male birds learn these songs by imitating a male tutor, usually their father, and the songs can be modified in adulthood in response to auditory feedback. Now, in *eLife*, Richard Mooney and colleagues at Duke University—including Kosuke Hamaguchi and Katherine Tschida as joint first authors—address the important question of how the sounds that birds hear influence the sounds that birds make (Hamaguchi et al., 2014). The findings have broader implications for our understanding of how sensory feedback is assimilated into complex motor programs in the brain, like learning to play a tune on the piano.

The circuits in the brain involved in producing birdsong are well defined (Figure 1). A ‘motor pathway’ is critical for song production and includes a brain area called HVC, which receives inputs from brain areas that process auditory information (Nottebohm et al., 1976; Hahnloser et al., 2002; Roy and Mooney, 2009). A different circuit, the anterior forebrain pathway (AFP), is critical for song learning. Important roles are played by nerve cells called HVCx cells that carry signals from HVC to the anterior forebrain pathway. The output of the AFP, a cluster of neurons called LMAN, sends signals back to the motor pathway: this area of the brain has also been identified as the source of the signals that modify song during vocal learning (Andalman and Fee, 2009; Warren et al., 2011; Charlesworth et al., 2012).

**Figure 1.**
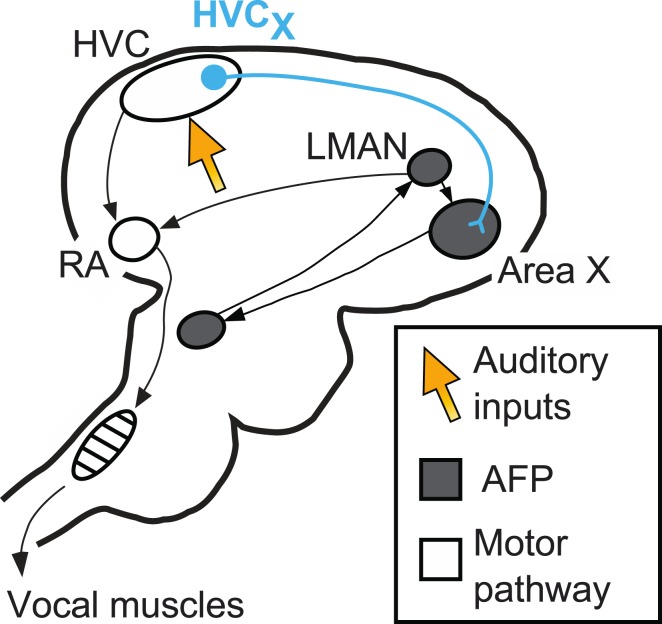
The neural pathways in the brain responsible for birdsong. A brain region called HVC sits at the interface between sensory input and motor output. It receives inputs (orange arrow) from the areas of the brain that process auditory information, and sends outputs to the vocal muscles (along the motor pathway) and also to Area X. Area X is part of the anterior forebrain pathway (AFP), which is essential for song learning in young birds. The output of this pathway, a cluster of neurons called LMAN, also sends signals to the robust nucleus of the arcopallium (RA), which is part of the motor pathway. Mooney and co-workers focus their attention on nerve cells called HVCx cells, which carry signals from HVC to the anterior forebrain pathway.

Neurons transmit via electrical signals that can be passed from cell to cell across gaps called synapses. When the electric potential of the cell membrane increases above a certain threshold, a voltage spike is produced. This is also referred to as the neuron firing. Variations in membrane potential that do not exceed threshold are known as ‘subthreshold’ activity.

HVC_X_ neurons fire when a bird sings, and the same pattern of firing is observed when a recorded song is played back to the bird (Prather et al., 2008). Because HVC_X_ neurons have both motor and sensory responses, and because they are connected to the anterior forebrain pathway, HVC_X_ neurons are a natural place to look when investigating how auditory feedback can alter song output. Previous studies have shown that the spiking activity (recorded with extracellular methods) of HVC_X_ neurons is not altered when birds are exposed to a distorted version of their own singing. Given that HVC_X_ neurons appear to be responsible for all auditory responses in the anterior forebrain pathway, this result seemed puzzling.

The Duke researchers re-examine this issue by using sharp electrodes to make intracellular recordings in freely moving singing birds. These technically demanding experiments employ a head-mounted microdrive connected to recording amplifiers via a lightweight wire tether (Long et al., 2010). That birds sing with this contraption on their heads, and that multiple long-duration recordings can be made, is a testament to both bird and experimenter.

The hypothesis being tested by the Duke group was that distortions of auditory feedback would alter subthreshold activity in the HVC_X_ cells as the bird sang. However, painstaking recording and analysis did not reveal any alterations, suggesting that the HVC_X_ neurons ‘ignore’ the distorted signals during singing. Although the hypothesis was disproven, this result is remarkable because these same neurons responded to auditory signals when the bird listened without singing. The Duke group also showed that changes in subthreshold potential in HVC_X_ neurons preceded song syllable onsets by tens of milliseconds, providing further evidence that synaptic inputs onto HVCx neurons convey motor-related signals during singing (Kozhevnikov and Fee, 2007; Prather et al., 2008). Mooney and co-workers therefore conclude that singing shuts off all auditory input to the HVC_X_ cells while still allowing motor signals to reach the AFP.

If HVCx cells are insensitive to auditory feedback, then how does this feedback alter both singing behaviour and the structure of HVC neurons? To explore this question the Duke team reopened the issue of how deafening (by removal of the cochlea) leads to atrophy of the inputs to HVC_X_ neurons (Tschida and Mooney 2012). This involved performing elegant in vivo imaging of single dendritic spines: dendrites are the long processes that extend from the body of a nerve cell, and spines are small needle-like structures that extend from the dendrites and receive synaptic inputs from other cells. Previously, lesion studies have shown that LMAN is required for the degradation of song that follows when deafening is used to remove auditory feedback (Brainard and Doupe 2000), and later it was shown that such deafening also causes atrophy of the spines on HVCx neurons (Tschida and Mooney, 2012). These observations led Mooney and co-workers to ask whether post-deafening spine changes were solely driven by direct inputs to HVC that convey auditory signals, or instead required signals from LMAN. It was found that damage to LMAN prevented deafening-induced changes in HVC_X_ neuron spines.

Taken together, the results of this study suggest that the HVCx cells are insensitive to real-time auditory information and that they are also downstream of the error-related signals that drive learning. A key challenge, therefore, is to determine how error information enters the anterior forebrain pathway. Mooney and co-workers suggest that dopamine signals from the midbrain are a likely candidate as they provide direct input to the anterior forebrain pathway without passing through HVC.

Neurons that are involved in both sensory and motor circuits, such as HVCx neurons, are particularly difficult to study. Activity in these circuits is both sensory and motor in nature, and sensory influences on neural activity can change dramatically across behavioural conditions. Studies such as this one by Mooney, Hamaguchi, Tschida and co-workers, which combine detailed functional analyses of identified cell classes with real-time manipulations of feedback, can reveal how complex interactions between sensory and motor signals shape the structure and function of the nervous system.
